# Artificial pneumothorax improves radiofrequency ablation of pulmonary metastases of hepatocellular carcinoma close to mediastinum

**DOI:** 10.1186/s12885-021-08223-7

**Published:** 2021-05-06

**Authors:** Taiyang Zuo, Wenli Lin, Fengyong Liu, Jinshun Xu

**Affiliations:** 1 Department of Interventional Oncology, Jinan Central Hospital Affiliated to Shandong University, Jinan, 250013 Shandong China; 2 Department of Interventional Radiology, Department of Interventional Ultrasound, Chinese PLA General Hospital, Beijing, 100853 Shandong China; 3 Department of Medical Ultrasound, Laboratory of Ultrasound Imaging Drug, West China Hospital, Sichuan University, Chengdu, 610041 Sichuan China

**Keywords:** Lung metastasis, Artificial pneumothorax, Mediastinum, Radiofrequency ablation, Hepatocellular carcinoma

## Abstract

**Background:**

To investigate the feasibility, safety and efficacy of percutaneous radiofrequency ablation (RFA) of pulmonary metastases from hepatocellular carcinoma (HCC) contiguous with the mediastinum using the artificial pneumothorax technique.

**Method:**

A total of 40 lesions in 32 patients with pulmonary metastases from HCC contiguous with the mediastinum accepted RFA treatment from August 2014 to May 2018 via the artificial pneumothorax technique. After ablation, clinical outcomes were followed up by contrast enhanced CT. Technical success, local tumor progression (LTP), intrapulmonary distant recurrence (IDR), and adverse events were evaluated. Overall survival (OS) and local tumor progression free survival (LTPFS) were recorded for each patient.

**Results:**

The tumor size was 1.4 ± 0.6 cm in diameter. RFA procedures were all successfully performed without intra-ablative complications. Technical success was noted in 100% of the patients. Five cases of LTP and 8 cases of IDR occurred following the secondary RFA for treatment. Slight pain was reported in all patients. No major complications were observed. The 1, 2, and 3-year LTPFS rates were 90.6, 81.2, and 71.8%, and the 1, 2, and 3-year OS rates were 100, 100 and 87.5%, respectively.

**Conclusion:**

Artificial pneumothorax adjuvant RFA is a feasible, safe, and efficient method for treatment of pulmonary metastases from HCC contiguous with the mediastinum.

## Background

Image-guided percutaneous local ablation has been widely used for the treatment of pulmonary metastasis, based on the high capability on accurate orientation of needles and real-time visualization of targets [1–5]. However, the minimal invasive thermal technique remains challenging for treatment of pulmonary metastasis abutting the mediastinum, as a result of the proximity to the heart or aorta. In 2007, Iguchi et al. reported that the primary technical effectiveness of radiofrequency ablation (RFA) was only 43% at 6 months postablation for treatment of 15 lung tumors contiguous with mediastinum [6]. Subsequently, studies on the efficacious improvement of RFA for treatment of lung tumors contiguous with mediastinum were rarely reported because of the insurmountable technique risk. Therefore, a safe and effective adjuvant method should be developed to reduce the risk of puncture and ablation for improvement of RFA efficiency of lung tumor contiguous with mediastinum.

Artificial pneumothorax is a standard technique that serves as an effective adjuvant for the clinical ablation of peripheral lung tumors [7, 8], which could be used for separation between the punctured needle path and normal tissues after gas administration into the pleural cavity [9]. It is suggested that the pulmonary tumors could be accurately separated from mediastinal structures during an artificial pneumothorax procedure. In that case, thermal damage to heart, aorta, or main nerves would be controlled, and pain caused by pleura heat could be effectively alleviated. In addition, the heat-sink effect of RFA could be reduced as well due to the artificial-induced space between tumors and vessels [10]. Therefore, this strategy could not only significantly reduce the risks of puncture and ablation, but also enhance RFA efficiency for complete ablation. Compared with the air administration to form artificial pneumothorax [9], CO_2_ is less common but displays good properties including high diffusion coefficient, inert reaction to RFA, and pure gas injection without unidentified substance [11]. Therefore, we investigate the feasibility, safety and efficacy of using artificial CO_2_ pneumothorax in this study for mediating RFA of pulmonary metastases contiguous with the mediastinum.

## Methods

### Patients enrollment

This study was approved by the institutional ethics committee of our hospital, patients diagnosed with pulmonary metastases contiguous with the mediastinum who underwent RFA were retrospectively investigated at our hospital from August 2014 to May 2018. The target lesions of all cases were confirmed clinically or pathologically as hepatocellular carcinoma (HCC). “Contiguous with mediastinum” was defined as the distance between metastatic lesion and mediastinum was less than 3 mm on CT or MR images.

The inclusion criteria for this study were: I) a single metastatic lesion contiguous with mediastinum in the unilateral lobe of lung; II) lesion size less than 3 cm in diameter; III) orthotopic HCC shown to be inactive without recurrence and intrahepatic metastasis as indicated by enhance imaging after surgical and (or) interventional therapy; IV) no increase in numbers of pulmonary lesions after 1–2 months of observation; V) patients refuse to accept surgical resection; and VI) Karnofsky performance status (KPS) greater than 70%.

The exclusion criteria were: I) patients with severe coagulation dysfunction including thrombocyte number < 30 × 10 [9]/L, international normalized ratio (INR) > 3.0, prothrombin time > 30 s, or prothrombin activity (PTA) < 40%; II) acute infection or chronic infection in acute phase; III) severe pulmonary hypertension defined as mean pulmonary artery pressure (mPAP) > 35 mmHg measured by cardiac catheterization and (or) Doppler echocardiogram; IV) severe pulmonary insufficiency defined as PaO_2_ < 60 mmHg, with and without PaCO_2_ > 50 mmHg; and V) implantation of cardiac pacemaker.

### Artificial pneumothorax adjuvant RFA procedure

All patients underwent contrast enhanced CT (CECT) of the chest prior to RFA in order to evaluate the anatomic relationship between lesions and peripheral cardiovascular structures. CECT was performed using the SOMATOM Force CT system (Siemens, Munich, Germany) with intravenous administration of Omnipaque (GE Medical, USA) as a contrast agent. Before the procedure, patients in a supine position received continuous electrocardiogram (ECG) monitoring and were hypodermically injected with 1% lidocaine. Then, the puncture point and needle track were determined by CT scan (MIYABI, Siemens, Munich, Germany). Subsequently, the biopsy needle was penetrated into the target in a direction away from the heart and vessels. When the procedure was accomplished, the biopsy needle was removed.

Thereafter, the artificial pneumothorax technique was performed using a 22-G puncture needle (NPAS-100, COOK, USA) with a blunt tip. The needle was inserted along the well-designed puncture proposal, which specifies insertion route, depth and angle. When the needle tip reached the edge of the pleura, the needle core was pulled out, 1–2 ml of saline was injected locally to form a water capsule using T-stopcock and tubes. Then, saline in the tube flowed into the cavity, and the water capsule disappeared gradually as the needle tip continued to be inserted and reached into the pleura cavity. Then, 100–500 ml of CO_2_ gas was frequently administered to separate the lung parenchyma. CT scanning was performed individually after injection of 100, 200, 300, 400, and 500 ml CO_2_ until the tumor was separated from the mediastinum and a feasible puncture path was established.

After the RF electrode was successfully inserted into the tumor based on the CT scanning assessment, percutaneous RFA (Model 1500, RITA Medical System, Mountain View, CA, USA) was performed at 60–70 W for 5–12 min at a rating temperature of 90 °C. During the process, an 18-G unipolar electrode (Uniblate) was chosen (the length of the needle was 15 cm and the maximum ablation range of a single electrode was 3 × 3 cm^2^). The ablation procedure was terminated when the ablation zone completely overlapped the target tumor and an ablative margin of 5–10 mm beyond the tumor boarder was achieved. Then, the RF electrode was withdrawn for coagulation of the needle track to avoid needle implantation and bleeding. During the procedure, 5–10 mg of morphine was intravenously injected for analgesia and sedation based on pain degree of patients. The ablation zone was defined as the pulmonary texture around the tumor that showed a circular exudation shadow with ground-glass appearance on CT imaging. After RFA, CO_2_ in the pleura cavity was aspirated with a 50-ml syringe and expelled through the T-stopcock until no additional gas could be aspirated.

### Follow up

CECT was performed to evaluate the therapeutic response of tumors at 1, 3, 6, and 12 months post ablation and at 6-month intervals thereafter. If incomplete tumor ablation was detected by CECT, secondary RFA was performed. According to the reporting criteria of image-guided tumor ablation [12, 13], technical success was defined as a tumor that was treated according to the initial protocol and was covered completely by the ablation zone. Local tumor progression (LTP) was defined as the appearance of new tumor foci at the ablative margin. Intrapulmonary distant recurrence (IDR) was defined as any occurrence of a new tumor foci in the lung.

During follow-up, adverse events (AEs) after ablation, local tumor progression free survival (LTPFS), and overall survival (OS) were recorded for each patient. AEs were classified in this study according to AE classification of the Society of Interventional Radiology [14]. The definition of major complication included moderate AE, severe AE, life-threatening or disabling event, patient death or unexpected pregnancy abortion. Mild AE was defined as minor complication. To assess the post-ablative pain, a visual analogue scale (VAS, a numerical rating scale: 0–10) was used based on previous publications: score 1–3 corresponding to slight pain, score 4–6 corresponding to moderate pain and score 7–10 corresponding to severe pain [5, 15].

### Statistical analysis

Statistical analysis was performed using SPSS software (version 22.0; SPSS Inc., Chicago, IL, USA). Categorical variables were described as numbers (percentages). Continuous variables were described as mean ± standard deviation (SD) or medians (range) according to the normality results using the Kolmogorov–Smirnov test. Survival was calculated by Kaplan-Meier survival analysis.

## Results

### Patients characteristics

**Fig. 1:**
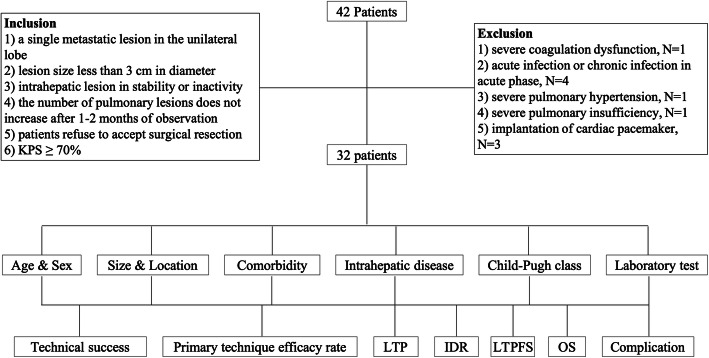
Graphical diagram of this study. KPS: Karnofsky performance status; LTP: local tumor progression; IDR: intrapulmonary distant recurrence; LTPFS: local tumor progression-free survival; OS: overall survival

The graphical diagram of this study is shown in Fig. 1. A total of 32 patients with 40 pulmonary metastatic tumors contiguous with the mediastinum were confirmed from HCC using biopsy and then accept RFA treatment during this study. Prior to RFA treatment, no other treatment options were performed in all patients. As shown in Tables 1, 62.5% (20/32) of patients were males, and 37.5% (12/32) of patients were females. The median age was 61 years old, ranging from 44 to 72 years old. The mean tumor diameter was 1.4 ± 0.6 cm with a median distance of 0.1 cm from the mediastinum, ranging from 0 to 0.3 cm. 87.5% (28/32) patients had cirrhosis, mainly due to viral hepatitis B or C (96.4%). In total, 56.2% (18/32) of patients were characterized as Child-Pugh classification A, and 43.7% (14/32) of patients were characterized as Child-Pugh classification B. The median model for end-stage liver disease (MELD) score was 12 with range between 6 and 22.

**Table 1:** Characteristics of the 32 patients with 40 pulmonary metastatic tumors contiguous with the mediastinum

Characteristics	Values, n (%)
Age (y), median (range)	61 (44–72)
Sex	
Male	20 (62.5)
Female	12 (37.5)
Pulmonary metastatic tumors (cm)	
Tumor diameter (mean ± SD)	1.4 ± 0.6
Distance from mediastinum, median (range)	0.1 (0–0.3)
Comorbidity	
Cirrhosis	28 (87.5)
Hypertension	12 (37.5)
Diabetes	10 (31.2)
Cardiovascular disease	6 (18.7)
Intrahepatic diseases	
Viral hepatitis B	20 (62.5)
Viral hepatitis C	7 (21.9)
Alcohol	2 (6.2)
Others	3 (9.4)
Child-Pugh class	
A	18 (56.2)
B	14 (43.7)
Laboratory data (mean ± SD)	
AFP (ng/ml)	88.7 ± 67.2
Total bilirubin (μmol/l)	19.1 ± 8.4
Albumin (g/l)	35.2 ± 6.7
Prothrombin time (%)	80.4 ± 18.3
Platelet count (10^9/l)	156 ± 48
Creatinine (mmol/l)	6.5 ± 3.2
MELD score, median (range)	12 (6–22)

*AFP* alpha fetal protein, *MELD* model for end-stage liver disease, *SD* standard deviation

### Therapeutic outcomes

**Fig. 2:**
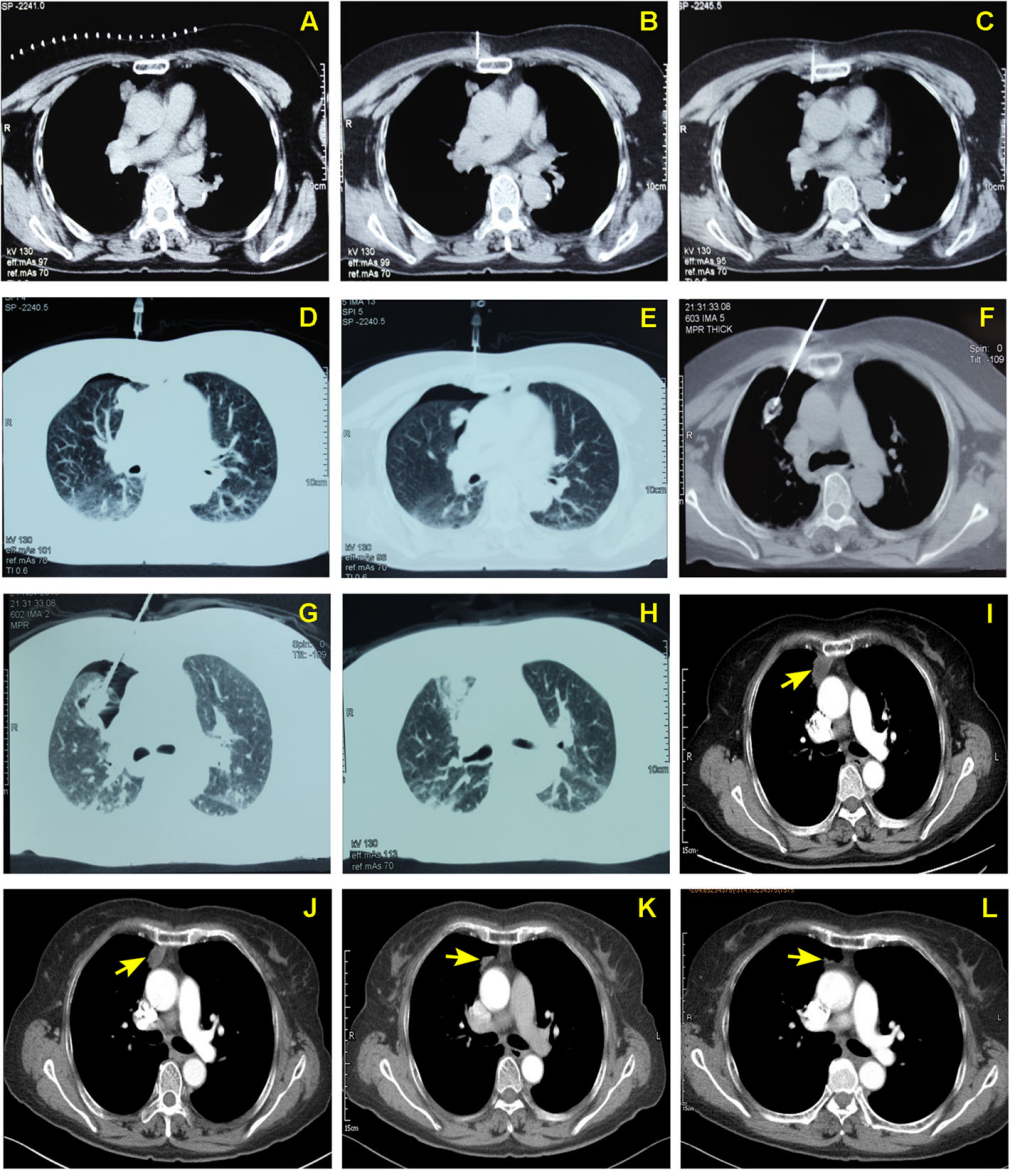
Artificial pneumothorax adjuvant RFA of pulmonary metastases contiguous to the mediastinum (a 67-year-old man with a metastatic lesion in the superior lobe forepart of right lung). Tumor size, 1.5 × 1.2 cm. **a** Before ablation, chest CT imaging was performed to evaluate the anatomic relationship between tumor and peripheral cardiovascular structures. **b** Subsequently, a 22-G needle tip was used to create a puncture that reached the outer edge of pleura for injection of 1–2 ml saline. **c** The needle tip entered into the pleura, and the saline in the tube flowed into the cavity. **d**-**e** CO_2_ gas was administered gradually with a syringe until the tumor was separated from the mediastinum. **f** CT image during RFA showed the electrode inserted into the tumor and located away from the mediastinum by proxy of artificial pneumothorax. **g** The ablation zone gradually increased following the RFA procedure. **h** After RFA, the pulmonary texture around tumor showed a circular exudation shadow with ground-glass appearance on CT image. **i** Contrast enhanced CT image 1 month after RFA showed no enhancement of the ablated tumor contiguous to the mediastinum. **j**-**l** The size of ablated tumor decreased gradually after RFA during follow up at 3, 6, and 12 months, respectively

**Fig. 3:**
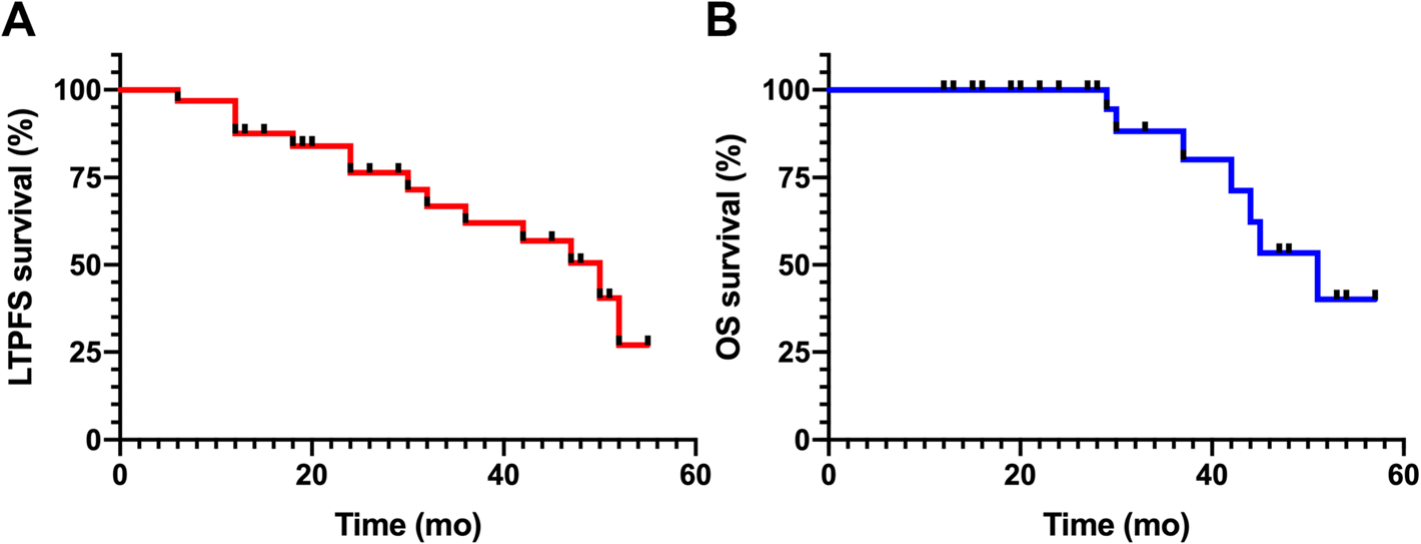
Overall survival (OS) and local tumor progression-free survival (LTPFS) curves during patient follow-up

RFA technical success was achieved in all patients after the average 267.5 ± 94.4 ml administration of CO_2_ was performed in each case. One of the most representative cases was presented in Fig. 2a-l. During the median follow-up of 29 months (ranging from 12 to 57 months), LTP occurred in 15.6% (5/32) of patients and IDR occurred in 25% (8/32) of patients (Table 2). Subsequently, the secondary RFA treatment were performed for the patients with LTP and IDR and technical success was obtained on CECT imaging assessment. The 1, 2, 3-year LTPFS rates after RFA were 90.6, 81.2, and 71.8%, and OS rates of 1, 2 and 3 years were 100, 100 and 87.5%, respectively (Fig. 3).

**Table 2:** RFA Outcomes of pulmonary metastases contiguous with the mediastinum (*n* = 32)

	Values, n (%)
Follow up (months), median (range)	29 (12–57)
Technical success	32 (100)
Local tumor progression (LTP)	5 (15.6)
Intrapulmonary distant recurrence (IDR)	8 (25)
Major complications	0
Minor complications	
Slight pain	32 (100)
Asymptomatic pneumothorax	10 (31.2)
Asymptomatic pleural effusion	4 (12.5)
Low-grade fever	12 (37.5)
General malaise	16 (50)

### Complications

No major complications were observed during and following RFA procedure, such as severe pneumothorax, aeroembolism, hemothorax, lung abscess, alveolar bleeding, and tumor seeding. No AEs were relevant to the proximity of tumors to the mediastinum, such as vagus/recurrent laryngeal/phrenic nerve injury, vessel or oesophageal injury, pericardial effusion, arrhythmia, and cardiac infraction. The overall complication rate was 100% in the 32 patients with 40 tumors. All of them were minor complications including slight pain of VAS score 1–3 (*n* = 32), asymptomatic pneumothorax (*n* = 10), asymptomatic pleural effusion (*n* = 4), transient postablation syndrome of low-grade fever (*n* = 12) and general malaise (*n* = 16), as shown in Table 2. All complications resolved after 1–2 days postablation without any treatment.

## Discussion

Artificial pneumothorax was first reported by Francini a century ago to treat pulmonary tuberculosis [16]. By injecting nitrogen between the parietal and visceral pleura, tuberculosis progression was suppressed by the reduction of blood flow and lymphatic reflux after pulmonary compression. During the past decades and enlightened by this report, artificial pneumothorax has been successfully performed for protection of chest wall [17], relief of chest pain [18], and biopsy of pulmonary or mediastinal tumors [19].

In this study, the technique of artificial pneumothorax was first validated as a feasible, safe, and efficient adjuvant method for RFA upon treatment of pulmonary metastases contiguous with the mediastinum. As shown in the results, technique success of RFA was 100% (32/32) without major complications under artificial pneumothorax intervention, and the 1, 2, 3-year LTPFS rates were 90.6 and 81.2%, and 71.8%, respectively, resulting in the valid treatment efficiency and effective protection of proximate mediastinal structures. Compared to 43% only of the primary technical effectiveness reported by Iguchi et al, these results were comparable to the overall technique efficacy of standard RFA of lung intraparenchymal tumors away from mediastinum [20, 21]. In general, our findings indicated that the use of artificial pneumothorax had safe and efficient local control for CT-guided RFA of lung metastasis contiguous with the mediastinum.

Given that 18-G needles are commonly used for gas administration in thorax, a potential complication for that procedure is mainly iatrogenic pneumothorax [8, 9, 22, 23]. To avoid damage to visceral pleura or pulmonary parenchyma, we used a 22-G blunt needle with negative pressure during the procedure and withdrew CO_2_ gas from the plural space after ablation. As a result, persistent pneumothorax or pleural hemorrhage with a chest tube drainage did not occurred.

In comparison with the air used before, the administration of CO_2_ to produce artificial pneumothorax in this study could be adsorbed more quickly by blood and reduce the risks of air embolism and injuries to normal tissues [24]. As a result, the blood oxygen saturations in all of the 32 patients were not influenced based on the ECG monitoring.

However, excessive CO_2_ in the pleural cavity can cause several adverse effects, including a) decrease in returned blood volume, increase in central venous pressure, and decrease in blood pressure; and b) impaired respiratory function and results in dyspnea, especially in the patients with poor pulmonary functions [25]. Furthermore, when excessive compression of lung parenchyma changes the electrical conductivity and heat conduction of RF, the ablation efficiency will be influenced, resulting in the damage enlargement of lung tissues [9]. Therefore, it is preferable to minimize the amount of CO_2_ injection during the process of artificial pneumothorax. Compared to the previously reported injection volume of 400–1400 ml [22], the 100–500 ml of injected CO_2_ in this study was more efficient to establish a safe puncture path between the tumor and mediastinum.

As known, resection is preferred for the treatment of pulmonary tumors, especially for the lesions contiguous with mediastinum. However, as to the patients with pulmonary metastasis, inoperability and intolerance are commonly coexisted because of old age, comorbidity, and operative suffering sentiment. Therefore, radiotherapy is the most common option for the treatment of pulmonary metastasis contiguous with mediastinum. Unfortunately, only 63% of 2-year OS rate was reported after 327 inoperable pulmonary oligometastases treated with stereotactic body radiotherapy (SBRT) [26]. Comparatively, Wah et al. reported that the estimated OS rates at 1 and 3 years were 96.7 and 74.7%, respectively, after 60 colorectal pulmonary oligometastases treated by RFA [27]. To the treatment of extrahepatic oligmetastases from HCC, Zhao et al. have reported that the 1-, 2-year OS rates were 91 and 70%, respectively [28]. Consistent with these results, 1-, 2-, 3-year OS rates of 100, 100 and 87.5% were found in this study for the pulmonary oligometastases contiguous with mediastinum, attributing to the benefits of artificial pneumothorax adjuvant technique.

Of note, the technique is non-indicated in some cases, including patients suffering from pleural adhesion or severe respiratory insufficiency. Limitations on this study was related to the fact that only 32 patients with 40 tumors were subjected to this method, owning to the focus is on the metastatic tumors from HCC. Further studies should explore the artificial pneumothorax procedure as an adjuvant for RFA to understand the full extent of clinical benefits.

## Conclusion

CT-guided RFA after artificially pneumothorax is a safe and effective method for the treatment of pulmonary metastases contiguous with the mediastinum. More samples should be examined to confirm the present results.

## Data Availability

The datasets used and/or analyzed during the current study will be available from the corresponding author (xujinshun@wch.scu.cn) on reasonable request.

## Abbreviations

RFA: Radiofrequency ablation
HCC: Hepatocellular carcinoma
KPS: Karnofsky performance status
INR: International normalized ratio
PTA: Prothrombin activity
mPAP: Mean pulmonary artery pressure
ECG: Electrocardiogram
LTP: Local tumor progression
IDR: Intrapulmonary distant recurrence
AEs: Adverse events
OS: Overall survival
LTPFS: Local tumor progression free survival
VAS: Visual analogue scale

## References

[1] Smith SL, Jennings PE (2015). Lung radiofrequency and microwave ablation: a review of indications, techniques and post-procedural imaging appearances. Br J Radiol.

[2] de Baere T (2011). Lung tumor radiofrequency ablation: where do we stand?. Cardiovasc Intervent Radiol.

[3] Hiraki T, Gobara H, Fujiwara H, Ishii H, Tomita K, Uka M, Makimoto S, Kanazawa S (2013). Lung cancer ablation: complications. Semin Intervent Radiol.

[4] Wei Z, Li Q, Ye X, et al. Microwave ablation or plus monochemotherapy in elderly advanced non-small-cell lung cancer patients. Minim Invasive Ther Allied Technol. 2021;30(2):106–14. 10.1080/13645706.2019.1678173 31621453

[5] Xu J, Wu H, Han Z, Zhang J, Li Q, Dou J, An C, Qi E, Yu J, Liang P (2018). Microwave ablation of benign breast tumors: a prospective study with minimum 12 months follow-up. Int J Hyperth.

[6] Iguchi T, Hiraki T, Gobara H, Mimura H, Fujiwara H, Tajiri N, Sakurai J, Yasui K, Date H, Kanazawa S (2007). Percutaneous radiofrequency ablation of lung tumors close to the heart or aorta: evaluation of safety and effectiveness. J Vasc Interv Radiol.

[7] Huang J, Hu Y, Mu X, Liao J, Wang X, Zhang H, Wang G (2018). Thoracic ultrasound versus artificial pneumothorax in complications of medical thoracoscopy-a propensity score matching analysis. Journal of thoracic disease.

[8] Hou X, Zhuang X, Zhang H, Wang K, Zhang Y (2017). Artificial pneumothorax: a safe and simple method to relieve pain during microwave ablation of subpleural lung malignancy. Minim Invasive Ther Allied Technol.

[9] de Baere T, Dromain C, Lapeyre M (2005). Artificially induced pneumothorax for percutaneous transthoracic radiofrequency ablation of tumors in the hepatic dome: initial experience. Radiology.

[10] Hocquelet A, Balageas P, Frulio N, Trillaud H (2015). Aggressive Intrasegmental recurrence of Periportal hepatocellular carcinoma after radiofrequency ablation: role of ablative technique and heat-sink effect?. Radiology.

[11] Belteki G, Lin B, Morley CJ (2017). Weight-correction of carbon dioxide diffusion coefficient (DCO2 ) reduces its inter-individual variability and improves its correlation with blood carbon dioxide levels in neonates receiving high-frequency oscillatory ventilation. Pediatr Pulmonol.

[12] Ahmed M (2014). Image-guided Tumor ablation: Standardization of Terminology and Reporting Criteria—A 10-Year Update. Radiology.

[13] Ahmed M (2014). Image-guided tumor ablation: standardization of terminology and reporting criteria--a 10-year update: supplement to the consensus document. J Vasc Interv Radiol.

[14] Khalilzadeh O, Baerlocher MO, Shyn PB, et al. Proposal of a New Adverse Event Classification by the Society of Interventional Radiology Standards of Practice Committee. J Vasc Interv Radiol. 2017;28(10):1432–7. 10.1016/j.jvir.2017.06.019 28757285

[15] Deib G, Deldar B, Hui F, et al. Percutaneous microwave ablation and Cementoplasty: clinical utility in the treatment of painful Extraspinal osseous metastatic disease and myeloma. AJR Am J Roentgenol. 2019:1–8. 10.2214/AJR.18.20386 30917019

[16] Garbarino MC, Cani V, Mazzarello P (2018). A century ago: Carlo Forlanini and the first successful treatment of tuberculosis. Lancet.

[17] Solomon SB, Thornton RH, Dupuy DE, Downey RJ (2008). Protection of the mediastinum and chest wall with an artificial pneumothorax during lung ablations. J Vasc Interv Radiol.

[18] Hiraki T, Gobara H, Shibamoto K, Mimura H, Soda Y, Uka M, Masaoka Y, Toyooka S, Kanazawa S (2011). Technique for creation of artificial pneumothorax for pain relief during radiofrequency ablation of peripheral lung tumors: report of seven cases. J Vasc Interv Radiol.

[19] Scalzetti EM (2005). Protective pneumothorax for needle biopsy of mediastinum and pulmonary hilum. J Thorac Imaging.

[20] Jiang B, McClure MA, Chen T (2018). Efficacy and safety of thermal ablation of lung malignancies: a network meta-analysis. Ann Thoracic Med.

[21] Hiyoshi Y, Miyamoto Y, Kiyozumi Y, Sawayama H, Eto K, Nagai Y, Iwatsuki M, Iwagami S, Baba Y, Yoshida N, Kawanaka K, Yamashita Y, Baba H (2019). CT-guided percutaneous radiofrequency ablation for lung metastases from colorectal cancer. Int J Clin Oncol.

[22] Lin ZY, Li YG (2009). Artificial pneumothorax with position adjustment for computed tomography-guided percutaneous core biopsy of mediastinum lesions. Ann Thorac Surg.

[23] Fujiwara H, Arai Y, Ishii H, Kanazawa S (2016). Computed tomography-guided radiofrequency ablation for sub-diaphragm hepatocellular carcinoma: safety and efficacy of inducing an artificial pneumothorax. Acta Med Okayama.

[24] Favelier S, Guiu S, Cherblanc V, Cercueil JP, Krausé D, Guiu B (2013). Transthoracic adrenal biopsy procedure using artificial carbon dioxide pneumothorax as outpatient procedure. Cardiovasc Intervent Radiol.

[25] Hill RC, Jones DR, Vance RA, Kalantarian B (1996). Selective lung ventilation during thoracoscopy: effects of insufflation on hemodynamics. Ann Thorac Surg.

[26] Shultz DB, Filippi AR, Thariat J, Mornex F, Loo BW, Ricardi U (2014). Stereotactic ablative radiotherapy for pulmonary oligometastases and oligometastatic lung cancer. J Thorac Oncol.

[27] Zhong J, Palkhi E, Ng H, Wang K, Milton R, Chaudhuri N, Lenton J, Smith J, Bhartia B, Wah TM (2020). Long-term outcomes in percutaneous radiofrequency ablation for histologically proven colorectal lung metastasis. Cardiovasc Intervent Radiol.

[28] Mu L, Sun L, Pan T, Lyu N, Li S, Li X, Wang J, Xie Q, Deng H, Zheng L, Peng J, Shen L, Fan W, Wu P, Zhao M (2018). Percutaneous CT-guided radiofrequency ablation for patients with extrahepatic oligometastases of hepatocellular carcinoma: long-term results. Int J Hyperth.

